# New York Odontological Society

**Published:** 1890-06

**Authors:** 

**Affiliations:** New York Odontological Society


### NEW YORK ODONTOLOGICAL SOCIETY.

THE New York Odontological Society held its regular monthly meeting, Tuesday evening, February 18, 1890, in the New York Academy of Medicine, No. 12 West Thirty-first Street.

The president, Dr. J. Morgan Howe, in the chair.

*Dr. Benjamin Lord.*—We were all so interested in the discussion of the paper at the last meeting that we entirely forgot to express our thanks to the essayist for his kindness; and I move you, sir, that a vote of thanks be tendered to Dr. McCausey, of Janesville, Wis., for his very interesting and able paper.

Dr. Lord's motion was carried, and the secretary was instructed to communicate the same to Dr. McCausey.

*Dr. Lord.*—It will be remembered also that Dr. H. C. Meriam sent here quite a number of files, of a new and improved pattern, for finishing fillings, and there was no mention made of his kindness; I move you that a vote of thanks be given to Dr. Meriam for the presentation of the files at the last meeting, and that the secretary be directed to report the same to Dr. Meriam.

Dr. Lord's motion prevailed.

INCIDENTS OF OFFICE PRACTICE.

*Dr. William Jarvie.*—Mr. President, I met with a case the other day that was so peculiar to me that I thought I would take a model of the teeth and present it to the society. The patient is a lady from fifty to fifty-five years of age, with all the upper teeth in good condition, and of hard, dense structure. The left central, for several years, has been gradually receding; not by erosion or attrition, but simply settling into the jaw. Some five years ago, it being then somewhat shorter than its fellow, the longer tooth was ground away and they were made both of the same length. Since that time there has been a very marked recession or shortening of the left central. It is the first case of the kind that has ever come under my notice, and I do not think it is a common occurrence. It is not caused by the occlusion of the lower teeth, as the lower centrals do not touch the upper centrals.

*Dr. N. W. Kingsley.*—How does Dr. Jarvie know that the other teeth have not elongated?

*Dr. Jarvie.*—That is a very pertinent question. I had never seen the lady until within the last month or so. If it is an elongation of the other teeth it is an elongation of the whole upper denture. It may be that the other teeth have elongated and this tooth alone remained in place. The other teeth are all firm.

*Dr. C. E. Francis.*—It would seem to be an elongation of the other teeth.

*Dr. C. F. W. Bodecker.*—Mr. President, among several cases of antrum disease that I have had lately there is one exceptional case which I would like to report. The patient is a lady between twenty-five and thirty years of age. She tells me that about six or eight years ago she had the left upper lateral incisor filled with gold, and that some weeks after the tooth began to ache. She went back to her dentist, then in the country, who advised her not to do anything with it. About two years ago there was a swelling about that tooth, which swelling extended back almost to her ear and up under the eye. She again applied to her dentist, who told her that he could not find anything the matter with her teeth; that they were sound and in good condition. Last May she was sent to me. I found none of the teeth decayed, but upon examining the parts above the first and second left upper bicuspids and the first molar, I found that the external plate of the antrum had been entirely dissolved. I made an incision a little above the first bicuspid root, into which I inserted a drainage-tube. One peculiarity was that I could not find any opening between the antrum and the nose, and on further examination I found that the antrum was a very abnormal one. Measuring from the front backward it was only about three-quarters of an inch long. It begins between the roots of the first bicuspid and the eye-tooth and goes back about as far as the root of the first molar; no farther. I sent the patient back to the gentleman who sent her to me, a physician of Hoboken, to see what he could do with the case. He sent her to Dr. Lange, the well-known surgeon, who examined the case, and was at first under the impression that the trouble was not in the antrum at all, but that it was some cyst which had developed between the two plates of bone, and that one of the plates had projected itself into the antrum. There are such cases on record. But against this theory there was the fact that the cavity was lined by a mucous membrane, which, with a cyst in such a position, is hardly probable. The patient was then sent to Dr. Delevan, an eminent specialist of diseases of the nose and throat, who tried to re-establish the osteum maxilla (opening between the antrum and the nose) by forcing a galvanic cautery needle through. You will remember that in the normal condition this wall is exceedingly thin, and can very easily be perforated by the galvanic cautery; but in this case Dr. Delevan could not get through. If there had been a cyst lying just within the antrum, this wall would not have been any thicker than usual, I should suppose. We do not know what to do with it; whether to take out the drainage-tube on the buccal surface, and leave it to nature to heal, or whether to go through in spite of the thickness of the wall of the antrum. I would like to have Dr. Atkinson, or any one, give me some advice. The antrum is now in an absolutely healthy condition, except that the osteum maxilla is closed.

*Dr. Jarvie.*—Why don't you let it alone?

*Dr. Bodecker.*—It is necessary to have an air-passage through the nasal cavities. I have a case similar to that, which has been in that condition for about nine years, which was also examined by Dr. Abbott. I proposed an operation, but the patient's family physician objected to it, and the case remains as it was.

*Dr. George S. Allan.*—Mr. President, if there is nothing else to be presented, I would like to read a short preamble and resolution, after which, I will, in a few words, give my reasons for offering them to the society.

WHEREAS, Drs. Heitzmann, Abbott, and Bodecker have promulgated certain theories as to the histological characteristics of the teeth, claiming the demonstration of the presence of a protoplasmic reticulum in both enamel and dentine, and as said theories are not generally accepted, and the demonstrations are questioned or doubted by other gentlemen of our profession who have devoted much time to histological investigation; therefore, be it

*Resolved,* That Drs. Heitzmann, Abbott, and Bodecker be inquired of, by the secretary of this society, whether they will co-operate with this society in an effort to have the truth known in regard to these disputed points, by obtaining the consent of three experts, to be chosen in a manner satisfactory to themselves and to the officers of this society, which committee shall have the right to examine specimens (slides) submitted, and be requested to report to the society at as early a day as practicable.

You all know how much interested I have been in this subject, and also how much interested the three gentlemen named have been, and how they have repeatedly stated their views and asserted them as proven beyond question, and in a manner that would almost throw doubt on the common sense or good judgment of any one not accepting them. Now, these views, if I understand them, fundamentally alter all our accepted ideas as to tooth-structure; and if they are accepted, we, as a consequence, must accept certain other theories as to the pathological conditions of the teeth. The one depends upon the other, and if one falls to the ground the other falls with it. Furthermore, if these views are accepted, they greatly militate against the acceptance of the theories that were lately so ably brought before the dental world by Dr. Miller, of Berlin. As you all know, the New York dentists are held responsible by the profession at large for their correctness. In other words, outside of New York City but little has been said about the theories referred to, and but little credence has been given to them, as I understand it. Travel where one will, it will be found that the New York dentists are held responsible for these theories. This being the case, if we are acting in a measure as godfather to these views, I think it is right, just, and proper that we should be able to go before the dental world with our reasons for accepting them, and our proofs of their reliability and worth ready at hand. Such an endorsement we can give only after receiving a favorable report from a committee such as this preamble and resolution call for. But if the committee reports unfavorably, then the gentlemen must seek elsewhere for support. I have doubted them from the beginning, but if it is necessary for me in the end to recant, and eat humble pie, no one will do it with a better grace than I will. But I must be satisfied that I am wrong first. It would be very easy to offer a resolution here calling for a committee of three or more experts to call upon Drs. Heitzmann, Abbott, and Bodecker, and ask them to show the slides; but such an examination would be simply worthless. An expert in the use of the microscope is only made by long and careful study; it is not a subject which could be committed to any one who has not made it a study and given it both thought and attention; therefore it seems to me wise and proper that this society, under the conditions, should take this step, both for the sake of the gentlemen who have so boldly thrust these views before us and for the sake of those who have taken the liberty to doubt them. I submit this preamble and resolution for the consideration of this society, hoping that such a committee will be appointed.

*Dr. Jarvie.*—Mr. President, this is a very delicate affair, and we want to give it due consideration. There is one thought that comes into my mind regarding the acceptance of the resolution; that is, the propriety of the words "we want the truth known." We do not want to hurt the feelings of anybody. It raises the question whether the gentlemen have told the truth. That is the only thing I would take exceptions to,—the wording of that line. I know Dr. Allan does not mean anything of that kind, but it struck me that possibly it might be given that interpretation. I think that otherwise it is a very proper thing.

*Dr. Allan.*—I simply want to know the facts of the case, or rather, I want the society and the profession to know what the truth is: whether a reticulum exists or not. It is not a question of the integrity or honesty of the gentlemen in the remotest degree. It is only a question of whether their slides show a reticulum. It is a mere matter of demonstration; and the question can only be decided by submitting the slides to gentlemen whose ability to decide such a question is beyond dispute.

*Dr. S. G. Perry.*—Mr. President, it seems to me that this is a very fair proposition. I read that resolution before it was presented here. It seems to be drawn in a good spirit, and I hope it will pass. I for one would like to have this question settled. It is a question of the minute structure of the teeth, and I do not know how it can be settled unless it is done by those who are truly experts in the matter.

Dr. Allan's preamble and resolution were adopted.

*The President.*—Gentlemen, I now have the pleasure of introducing to you Dr. Edward S. Peck, of this city, whose name, no doubt, you are familiar with. Dr. Peck will read a paper entitled "Reflex Ocular and Facial Symptoms of Nasal Disease." (For paper, see page 321.)

*The President.*—The subject of Dr. Peck's very interesting and able paper is before you, gentlemen, for

DISCUSSION.

*Dr. C. E. H. Phillips.*—Mr. President and gentlemen, I am sure every one must feel, as I do, very grateful to Dr. Peck for the care and time he has given to the preparation of this paper, and I think it will call the further attention of gentlemen in his specialty, as well as that of the dental surgeon, to this important subject of reflex irritation. In the transactions of the Odontological Society of Great Britain for November, 1883, there is a rather lengthy paper which bears strongly upon this subject, and which must be of great interest to any one who has not already seen it. It is by Henry Power, of London, who is ophthalmic surgeon in St. Bartholomew's and Westminster Hospitals. His paper is on the relation of dental lesions to diseases of the eye. He has consulted authorities for some fifty or one hundred years back, and cites a number of cases of his own and other authorities, mentioning cases where the eye was affected by disease of the antrum and by difficult dentition. He regards the subject of reflex irritation as of great importance. His paper is in three sections,—reflex irritation affecting the striated and unstriated muscles, reflex irritation affecting the cornea, and reflex irritation affecting the optic nerve and the retina,—all of which have some very fine points. He states that one of the commonest forms of facial disturbance induced by these diseases is the failure or loss of power of accommodation. In many cases of ocular disease depending upon dental lesions great improvement was found after the extraction of the diseased teeth, and in many cases a cure resulted from this treatment. Both his own cases and those of other authorities are mentioned. There are two which seem to be rather important. One is a case where loss of sight occurred for thirteen months and was restored by the extraction of a tooth the roots of which penetrated the antrum.

Another rather curious case of intermittent blindness, it might be called, was due to a carious molar tooth into which food was crowded. When the food became impacted in this tooth, loss of sight was the result, and when the tooth was opened, sight was restored, and was permanently restored by the extraction of the tooth.

The *Medical Record*, of September, 1888, mentions a case of amaurosis arising from a carious tooth. It is a case of Dr. Rivas, quoted from a French journal, the case of a woman thirty years of age who was wholly blind in one eye. She suffered from a carious tooth on the same side; the extraction of the tooth restored her sight, and she had no more trouble in that way.

I would like to state a case in my own practice,—a lady who had been treated nearly a year for ear trouble and slight facial neuralgia. She resided in New Jersey, and had been treated by her local physician without beneficial results. She consulted, in Jersey City, a friend of mine who is a specialist for the eye and ear, and he made a careful examination without being able to find the cause of trouble. When she came to me, I made a thorough examination of the mouth. She had a number of teeth filled, but the work seemed to have been well done, and she complained of no trouble at all in the mouth; no pain about the teeth. I was about to return her to the physician with the statement that her mouth and teeth were in good condition, when, to be more thorough, I took a little wedge of pine wood and placed it between the teeth, requesting her to bite on it as it was passed around from one side of the mouth to the other. When biting on the wedge placed over the inferior twelfth-year molar on the right side, she spoke of "a little different feeling" in that part of the mouth from what she noticed elsewhere. It was not pain, but a different feeling. On taking the handle of a mouth mirror and tapping on the teeth, the moment I touched that tooth she repeated that it felt different from the other teeth. I found quite a large and nicely-appearing gold filling in this tooth, which I told her I would like to remove. I removed the filling, and as I took the last piece of gold out there was an odor of decomposition, and on cutting into the tooth a little farther I discovered it to be dead. In one of the root-canals there was a small quantity of pus. I told the lady I believed this tooth was the cause of the ear trouble, and I requested her to have it treated, which she very willingly did. I treated it a number of times, removed all the decomposed pulp, and afterwards filled the tooth. The neuralgic trouble diminished immediately on opening the tooth, and there has been no recurrence of it since the tooth was filled. There was no facial neuralgia or trouble in the ear while the tooth was under treatment, nor since.

*Dr. A. B. Norton.*—Mr. President, the preceding speaker has alluded to cases of blindness having been cured by extraction of the teeth. I would like to say that not only are there cases on record of vision having been restored by the extraction of teeth, but cases also have been reported of vision having been *lost* after the extraction of teeth. In another disease, which I do not think Dr. Peck mentioned in his paper, there is a case reported by Galezowski, a case of exophthalmia of one eye, with optic neuritis and complete blindness, in which the patient suffered a great deal of pain, which, with the exophthalmia, was relieved entirely by the extraction of a carious molar. Vision was, of course, destroyed from atrophy of the optic nerve, and was not restored.

A year or two ago I had a rather peculiar case of exophthalmia of one eye only; and, not being satisfied in regard to the condition of the teeth, I referred the patient to Dr. Howe, who reported the teeth as being in good condition and not the cause of the eye trouble. The patient was then referred to a neurologist, who found that the exophthalmia was due to some cerebral trouble.

I have recently seen a report of one or two cases of headache in which an oculist had treated the eye with correcting lenses without giving relief. Attention was then directed to the teeth, and some gold and amalgam fillings were found, and the headaches were entirely cured by taking out the amalgam fillings. The oculist, in reporting the cases, only made the assumption that *possibly* the headaches were due to the fact that, from mental or physical over-exertion, the saliva had become acid, and the acid saliva had served to set up an electrical action between the gold and amalgam fillings in the teeth.

I would like the opinion of some of the gentlemen present as to the theory suggested by the physician reporting these cases.

*Dr. Charles D. Cook.*—I would like to ask whether the pulps were dead in the teeth in which the amalgam fillings were, or whether the teeth were sound and healthy.

*Dr. Norton.*—The cases as reported stated that there was ulceration at the root of the amalgam-filled tooth.

*Dr. Cook.*—I should doubt the assumption, then, of sufficient electrical action being set up to cause headache or any other pain. The fact that the tooth was ulcerated was sufficient, without any other consideration, to account for the disturbance. I think dentists generally will bear me out in that conclusion.

*Dr. Bodecker.*—I am much pleased with this very interesting paper, and will mention a few cases of reflex action, although they do not directly apply to the subject. The first is one of a lady who some years ago came to me suffering with pulpitis of the left upper lateral incisor. As soon as I opened into the pulp-chamber from the lingual surface she complained of a terrible pain in her fingers. I was told that she had been to Wiesbaden two years in succession for relief from rheumatism in the corresponding arm and shoulder. I destroyed the pulp. Two days after that I saw the lady again, and she told me that the queer sensation which she had had in that arm was gone. I have seen the patient since, but the pain in her extremity never returned. After the pulp had been removed from the tooth it was immediately placed in a very weak solution of chromic acid, and afterwards embedded, cut, and prepared for microscopic examination, revealing numerous pointed speculæ of bone, which had developed in the pulp tissue, and a few of them between the bundles of medullated nerve-fibres.

Another case of a very peculiar character, which I have shown several times at clinics, was that of a lady of about twenty-five or twenty-eight years of age, who had a swelling of the left side of the lower jaw, which always came during the menstrual period, and remained there without any accompanying pain whatsoever. She had been to two other dentists, and they each took out one of the molars. First the wisdom tooth was removed, then the first molar. When I saw the lady I thought probably it was an abscess of the second molar, which was perfectly sound and healthy. I attempted to drill into it and found it to be alive. I immediately stopped the drill-hole with gutta-percha. I treated it without any success whatsoever. Dr. Atkinson saw the case at one of the clinics, but did not know what to make of it. At that time Dr. Louis Waldstein lived in my house, and he examined the case and inquired into it very minutely. I saw him last year, when he told me he had solved that mystery. He found that the swelling originated from some uterine trouble, which has since been cured, and the swelling has never returned.

*Dr. Littig.*—As the case that Dr. Peck last reported was seen by a number of the gentlemen here, and there was considerable consultation over it, I would like to state that my impression is that instead of the lateral tooth being broken off it was both the centrals. Of those gentlemen who examined the case some were of the opinion that the cause of the neuralgia was remote from the teeth, and others seemed to think it was due to one of the pulps of the broken teeth. Treatment was undertaken; I drilled into the tooth a certain distance and treated it, and that seemed to give temporary relief. The patient was very anxious that I should destroy the pulp of the tooth, which was done. I took out the pulp, and for a few weeks he said he thought he was better; but soon the pain returned with renewed vigor. The disturbance was more general, and the gentleman was very ill, for several weeks in his bed. The neuralgic pain continued until the operation was made that Dr. Peck has spoken of. Since that time there has been no trouble whatever. I only state the facts of the case here so that those who saw it may know the result of the case which they had under consultation.

*Dr. George Howe Winkler.*—Mr. President, I think that neuralgia of the face and head are caused by dental lesions about nine times out of ten; and the exhibitions are so varied that we are oftentimes unable to discover the true cause. A friend of mine consulted me once in regard to one of his patients who had suffered for two or three years with attacks of neuralgia of the eyes, so violent as to protrude the eyeballs from the sockets. She was scarcely free from neuralgic pains about the eyes for forty-eight hours at a time. I examined the case, and found two incisors with devitalized pulps, the other incisors having been lost. The cuspids were almost entirely denuded of their enamel, and at the margin of the gum all around they were exquisitely sensitive to the touch of an instrument or tooth-brush. I advised the immediate extraction of the two dead teeth, which were somewhat loose, and that the cuspids be retained. The other teeth had been lost, and she had only these four teeth. In order to relieve the exquisite sensitiveness of these teeth, I advised the making of a delicate platinum band to cover each denuded surface. My friend extracted the two dead teeth and let the patient rest a few days. She was relieved for three or four days; then the neuralgia returned with its usual intensity. He carefully adjusted these platinum bands around the teeth, and for three weeks she did not have a single twinge of neuralgia. I lost sight of the patient, but for the three weeks that she was under my observation these sensitive surfaces were comfortable, and she was entirely relieved from neuralgic pain after having suffered almost two years.

*Dr. C. A. Woodward.*—Mr. President, I have a case which is somewhat in the line of the subject to-night. Some four months ago a lady, thirty-nine years of age, came to me suffering with a discharge of pus from around the back part of the last tooth in the lower jaw. I examined her mouth and noticed the absence of the wisdom tooth. As she was quite sure it had never been extracted, I concluded that a retarded wisdom tooth was causing the discharge of pus. I burned away the gum quite freely, then burred away the alveolar process from around the wisdom tooth, when I found the tip pointing through the process. From the time I burred away the bone freely from around the tooth the pus stopped discharging. I dismissed the patient, thinking she would have no further trouble. She returned three weeks ago with a similar condition on the other side of the jaw. I came to the conclusion that it was caused by the other wisdom tooth, and I began to treat it in the same way. This was about two weeks ago. I saw the lady but twice, when she stopped coming; but to-day she came in and said I had spoken to her about retarded wisdom teeth sometimes causing a great deal of trouble, and she then told me that she had a large swelling on both sides of the lower part of the neck just over the thyroid gland. She informed me that she was under treatment for that swelling by one of the most renowned surgeons of New York without obtaining any relief; and she had noticed that when the teeth were troubling her most the swelling of the gland would be increased. I have but very little doubt that those retarded wisdom teeth are the cause of the swelling of the glands, which she considers very serious, having been told it might produce death if it went on, or at any rate that the gland must be removed.

*The President.*—I hope Dr. Woodward will report on that case at a future time.

*Dr. Woodward.*—I shall be glad to do so.

*Dr. George S. Norton.*—Mr. President, it seems to me that the subject of nasal reflex action is a little too high for the dentist and a little too low for the oculist. The discussion seems to have wandered from the subject of the paper. I have very little to do with the throat and nose, devoting my time to the eye exclusively. In my experience of twenty years I have not found that disease of the nose very frequently produced ocular disturbance from reflex irritation, though it does occasionally. I do sometimes find, however, that the eye is affected from the disease extending to the lachrymal ducts, causing partial closure of the lachrymal ducts and in that way affecting the eye. This subject has been elaborated somewhat extensively within the past two or three years, and within the last two months an article has been published at Paris, France, by Professor Galezowski, in which three or four cases are described in which symptoms simulating glaucoma were produced by obstruction of the lachrymal ducts, and which were relieved by probing of the ducts.

I have also occasionally found inflammation of the conjunctiva dependent upon reflex irritation from the nose and from catarrh involving the lachrymal apparatus.

The discussion has reference especially to the teeth, and that is a subject, I suppose, of prime importance to those present. In the first place I would say that I believe nasal disease more often causes trouble in the ear than it does in the eye. We also quite frequently find cases in which dental lesions cause inflammation of the ear, but not so frequently disturbance of the eye.

Turning to the teeth, there was one case that appeared in my practice four or five years ago which taught me that disease of the teeth may produce very severe trouble with the eye. It was a case in which I had operated for cataract. It was a perfectly smooth operation; nothing about it which should have caused any subsequent trouble. The next day I found that the man had suffered intense pain in an upper molar tooth on that side, and I then found for the first time that he had a decayed tooth. He had suffered all night from the pain, and it produced a most severe inflammation of the eye, and the eye was lost. That has made me very careful to question the patient before operating to ascertain if there are any decayed teeth that may cause trouble.

*Dr. E. A. Bogue.*—I would like to take the position of a questioner. The gentleman just behind me made reference to nasal reflex being too high for the dentist and too low for the oculist; and yet Dr. Sexton, whom I have not the honor of knowing, published an article, or two or three, on the subject of dead teeth in the mouth. That subject was brought to my mind by a remark of our friend who has just seated himself,—"disease of the teeth." I should very much like to know what he means by disease of the teeth. I have been trying for four or five years to find out what Dr. Sexton meant by dead teeth in the mouth. Indeed, from the depths of my ignorance, I believe that if there were any dead teeth in the mouth they would be expelled. I had supposed the symptoms of glaucoma might be produced by irritation almost anywhere along the track of the fifth pair of nerves. That those symptoms may disappear as readily as they arise, and that they may be caused by either one of three definite conditions which we as dentists are called upon to treat, may be readily believed. Those three diseases, or three manifestations of the same disease, perhaps, are as follows: First, if the limy matter which is deposited around the necks, and oftentimes around the roots, of teeth, is allowed to accumulate, it may produce a small abscess which, for the time being, sometimes a day, sometimes two days, will produce considerable pain. I know, for I have tried it. Secondly, exposure of a dental nerve or pulp. A good big molar tooth has something like two hundred thousand nerves in it, as nearly as we can find out, but it has only one pulp. If those nerves, or the peripheral extremities of the dental nerves, are exposed, they give pain, just as exposed nerves do elsewhere in the body. That pain, however, I believe, is not shown in reflected symptoms that would be taken cognizance of by gentlemen practising other branches. The third cause of pain from the teeth is pericementitis, and that very rarely takes place except as a complication with some of the causes of an abscess. That is a condition, as I understand it, which has called forth Dr. Sexton's paper, and which has called forth a good deal of interest, and possibly expression, of practitioners in other branches in regard to reflex action. This condition of abscess after the pulp of a tooth has died is a condition which almost always involves pressure. It always involves pus formation, and that formation of pus, existing rather deeply seated within bones which may or may not be spongy ones, causes pressure upon the dental nerve, and thus a reflection appears there or elsewhere. Necrosis I do not want to go into now. If it be true that exposure of the pulp of a tooth occasionally causes pain, which might give reflex action and confound us when we are making a diagnosis, and if it be true that abscesses arise from the decomposition of a pulp from which life has departed, it is time that we all look carefully into it. A dentist once consulted me as to what should be done with an abscessed tooth, and in that individual case I told him to drill in its full length and let out the pus. The next I heard of him he was drilling into teeth right and left. He was utterly unable to discriminate between teeth with living pulps and teeth with dead pulps. I have within a year seen a patient whose living pulp he had drilled into for the purpose of relieving pain.

*The President.*—Did Dr. Bogue say he was a dentist?

*Dr. Bogue.*—I beg your pardon, but I have again to expose my ignorance. He said he was. He has a sign out.

This desultory sort of talk, in which we wander a little from the subject, will probably allow me to allude to three instances. The first was a lady, who grew thin and anæmic, and was unable to nurse her child, six or eight months old. I insisted upon the loss of a central incisor. She insisted upon keeping it. The tooth was finally extracted, and she came three weeks afterwards to make her thanks, being in increased flesh and good appearance and the child well nourished.

*Dr. Lord.*—What was the condition of that tooth?

*Dr. Bogue.*—It was a pulpless tooth and had an abscess from that cause, and besides that the cementum was saturated with the products of abscesses for many years.

The second case was a lady who was sent to a physician for treatment of neuralgia of the neck. The physician examined her carefully and told her to consult a dentist, and sent her to me. I sent her back again. She once more returned to me. I then set to work with all the appliances I had. Finally I suspected a right lateral incisor, which she told me was filled ten years previously by Dr. Dwinelle, and had never since been operated upon. I concluded that tooth was the offender, and it was so proven. On opening it pus was found in considerable quantity.

The third case seemed to me a most unusual one. A lady presented herself for the purpose of having two or three artificial teeth put in. She declined my advice and went elsewhere. In a year she returned, because she had suffered constant pain. She had been to seven different men. I then found that the constant strain upon the mental faculties from the condition of the teeth had so upset the patient that when the apparatus came to be adjusted it was almost useless. It has resulted in a condition which four or five surgeons have pronounced tetanic. One after another, she has lost every tooth in her mouth, and her mental faculties have been so disturbed that she has once been arrested as being *non compos mentis.*

*Dr. George S. Norton.*—I have just one word to say. When the gentleman who has just preceded me criticises any statement that I have made in relation to the teeth, I will acknowledge my ignorance of the subject. But when he makes the statement, or infers that glaucoma is often dependent upon reflex irritation of the fifth pair of nerves, or reflex irritation in general, I must question it most decidedly.

*Dr. Bogue.*—I said the symptoms of glaucoma.

*Dr. Norton.*—I question that. That is a theory which I believe has long since been exploded. Years ago it was held that glaucoma was dependent upon reflex irritation, but now it is acknowledged by almost all specialists that glaucoma is a local disease in the eye, and that the reflex symptoms of neuralgia are simply dependent upon the glaucoma, not that the neuralgia is a cause of the glaucoma. That is the theory up to our present knowledge on the subject.

*Dr. Bogue.*—I desire to make my thanks for the correction, for that is valuable. It enables us also to bring our work down to the point. As I understand it, pain which proceeds from the teeth has a definite cause, and one can almost always find it. Exostosis I forgot in my enumeration of the three or four different causes of dental pain. We may not always be able to detect that, yet we find that the cause of the pain is local, although it may be that the symptoms appear here, there, and elsewhere; are, in fact, reflected.

*Dr. Dwinelle.*—Mr. President, I had supposed that glaucoma was induced by constriction of the optic nerve as it passes through the orbital foramen. I cannot say much as to the theory, but I remember that in my own practice I had several cases of glaucoma which have been manifestly influenced by treatment of the teeth. One very dear friend of mine was to have been operated upon five years ago; the doctors insisted upon it, stating that he could not retain his sight more than six or eight months longer at the utmost. But I felt encouraged otherwise. I felt that reflex action might have something to do with it, and that was the case. I was vigilant in looking after his teeth and his general health; and it is sufficient to say that perhaps our best authorities in reference to the treatment of the eye sometimes make mistakes in their diagnosis. The patient constantly improved, and his symptoms are certainly no worse now than they were four or five years ago. I believe that the operations I made upon the teeth have been exceedingly effective, and very likely have been the means of saving his sight. At any rate, the relief they gave him has been most manifest and permanent up to date.

In reference to reflex action, I think that we, as dentists, see constant manifestations of it. One familiar illustration of it is that while we are operating upon the upper teeth, the lower ones, which may be perfectly sound, sympathize with them through reflex action, and become subject to severe pain. We see it also in subduing sensitiveness of the teeth by applying caustics to the cervical regions of the teeth; immediately upon the application of the caustic to the teeth above, pain will immediately be felt below.

Referring to exostosis, we are continually witnessing most manifest demonstrations and illustrations of reflex action from exostosis affecting not only the ear and the nasal passages, but the eye as well. I remember an instance now that came under my observation, where a person had a very bad case of exostosis of the first left superior bicuspid; she became nearly blind in the eye corresponding to it. She was impressed with the idea that it proceeded entirely from that tooth. I was reluctant about removing it, for I had already placed an artificial crown upon it, and was desirous of retaining it. I believe that Europeans say American ladies are distinguished by having lost their superior bicuspid teeth; at any rate, they are careless about losing them. But in this instance, when I removed this tooth, very reluctantly, I found a marked case of exostosis. The extraction gave her immediate relief, and she has already nearly recovered her eyesight. Not only was the eye affected internally, but the conjunctiva and the external eye generally. The first cloudy indications of cataract were manifest to a marked degree. All those unhappy symptoms subsided. It is nearly a year since the operation, and all indications are favorable to date.

I am very glad that the subject of reflex action has been introduced to our attention to-night, and I wish to thank the essayist personally for it. The nasal passages are very materially affected by the teeth, and *vice versa.* Any interference with, or obstruction of, the nasal air-passages is a very important matter, and we ought to take it into consideration in a way that we have not heretofore. It is a field well worthy of patient investigation. Obstruction or interference with the air-passages has much to do with the malgrowth and deformities that continually arrest our attention. The reflex action and mechanical effects upon the teeth and jaws, from *adenoid vegetations* in children, thereby closing the nasal passages and compelling mouth-breathing exclusively, is a subject which as dentists should merit our most serious attention.

It is well known that the *naso-pharyngeal cavity* obtains but small recognition from our profession, whereas, it is often the almost exclusive region from which the most serious deformities of the teeth and jaws have their origin.

Dr. Woodward moved a vote of thanks to Dr. Peck for his very interesting paper. Seconded by Dr. Perry. Amended by Dr. Bogue to include the gentlemen who participated in the discussion. Adopted as amended.

Adjourned.

S. E. Davenport, D.D.S., M.D.S.,
*Editor New York Odontological Society.*

